# Comparison of the current AJCC-TNM numeric-based with a new anatomical location-based lymph node staging system for gastric cancer: A western experience

**DOI:** 10.1371/journal.pone.0173619

**Published:** 2017-04-05

**Authors:** Gennaro Galizia, Eva Lieto, Annamaria Auricchio, Francesca Cardella, Andrea Mabilia, Anna Diana, Paolo Castellano, Ferdinando De Vita, Michele Orditura

**Affiliations:** 1 Division of Surgical Oncology, Department of Surgical Sciences, University of Campania 'Luigi Vanvitelli', School of Medicine, Naples, Italy; 2 Division of Medical Oncology, "F. Magrassi" Department of Clinical and Experimental Medicine and Surgery, University of Campania 'Luigi Vanvitelli', School of Medicine, Naples, Italy; National Cancer Center, JAPAN

## Abstract

**Background:**

In gastric cancer, the current AJCC numeric-based lymph node staging does not provide information on the anatomical extent of the disease and lymphadenectomy. A new anatomical location-based node staging, proposed by Choi, has shown better prognostic performance, thus soliciting Western world validation.

**Study design:**

Data from 284 gastric cancers undergoing radical surgery at the Second University of Naples from 2000 to 2014 were reviewed. The lymph nodes were reclassified into three groups (lesser and greater curvature, and extraperigastric nodes); presence of any metastatic lymph node in a given group was considered positive, prompting a new N and TNM stage classification. Receiver-operating-characteristic (ROC) curves for censored survival data and bootstrap methods were used to compare the capability of the two models to predict tumor recurrence.

**Results:**

More than one third of node positive patients were reclassified into different N and TNM stages by the new system. Compared to the current staging system, the new classification significantly correlated with tumor recurrence rates and displayed improved indices of prognostic performance, such as the Bayesian information criterion and the Harrell C-index. Higher values at survival ROC analysis demonstrated a significantly better stratification of patients by the new system, mostly in the early phase of the follow-up, with a worse prognosis in more advanced new N stages, despite the same current N stage.

**Conclusions:**

This study suggests that the anatomical location-based classification of lymph node metastasis may be an important tool for gastric cancer prognosis and should be considered for future revision of the TNM staging system.

## Introduction

Gastric cancer is still a worldwide major leading cause of cancer death [1]. Despite recent advances obtained with multimodality therapy, including chemo and radiotherapy [2–4], prognostic accuracy remains a key issue to define treatment [5,6]. The TNM classification is the most important tool for treatment planning in oncology and for assessing patient prognosis [7]. The current staging system for gastric cancer is represented by the seventh edition of the American Joint Committee on Cancer (AJCC), where the extent of lymph node (LN) metastasis (N stage) is represented by a numeric-based system [8]. The last three editions differed from the previous ones, where the N stage was defined according to the anatomical location of involved lymph nodes [9–12]. The numeric-based system was introduced because it appeared to be considerably simpler and basically acceptable [13,14]. However, this system entails substantial limitations, mainly represented by lack of information on both the anatomical extent of LN dissection and involvement of proximal or distal nodes, which are known to be able to affect outcome [2,15,16]. In addition, discrepancies in survival rates among different N stages and within the same stage have been sometimes observed [5,13,17].

A new intuitive and simple N and TNM staging system, based on the location of LN involvement, has been recently proposed by Choi et al [18]. The new staging system was shown to have improved prognostic performance, thus suggesting it could be a reliable alternative to the current numeric-based system. However, as stated by the same authors, validation by Western investigators was deemed essential in order to allow global consideration of this staging system in gastric cancer.

Therefore, we reclassified our series of gastric cancer patients and analyzed the predictive capability and prognostic performance of the two competing staging systems, in order to determine whether the new proposed staging system could also be reliably used in Western patients.

## Methods

### Patient cohort

Four hundred twenty eight patients undergoing gastrectomy for histologically confirmed gastric adenocarcinoma between January 2000 and December 2014 were reviewed. Patients with adenocarcinoma of the esophagogastric junction (73 cases), which is distinguished from stomach cancers in the 7th AJCC edition [10], patients receiving neoadjuvant chemotherapy (42 cases), or undergoing non-radical surgery (namely, macroscopic or microscopic tumor residual or positive margins—17 cases), and patients who experienced postoperative death (12 cases) were excluded. Ultimately, a total of 284 patients were considered; age ranged from 29 to 84 years (median 63, interquartile range: 54–72 years). All patients were followed-up and underwent 5-fluorouracil plus oxaliplatin-based adjuvant chemotherapy, if appropriate [19]. No patient was lost to follow-up and it was complete by March 31, 2016. All patients gave written consent to personal data processing and the study was approved (certification number 48/2016, March 18, 2016) by the Department of Surgery of the Second University of Naples. Disease-free survival (DFS) rather than overall survival was preferred as a reliable and clinically meaningful outcome. DFS warrants earlier presentation of data because events occur earlier than death, thus limiting bias related to short follow-up times. Besides, DFS has recently been shown to be an acceptable surrogate for overall survival in oncologic diseases, including gastric cancer [20–22].

### Nodal dissection and classification

In agreement with Japanese guidelines [23], all patients underwent D2 lymphadenectomy, or, more recently, so called D1+ lymphadenectomy that avoids spleno-pancreasectomy without affecting oncological outcome [6,24,25]. According to Choi et al [18], each cancer was reclassified according to the anatomical location-based system of LN metastases. Briefly, LN groups were divided into perigastric and extraperigastric (EP) nodes. Perigastric nodes were further divided into nodes along the lesser curvature (LC: nodal groups no. 1, 3, and 5) and nodes along the greater curvature (GC: nodal groups no. 2, 4sa, 4sb, 4d, 6, and greater omentum). Finally, nodal groups from 7 to 12a were classified as EP. New N stage was defined as follows: new N0, no LN metastasis; new N1, positive LN in only one of three groups (LC, or GC, or EP), regardless of number of involved lymph nodes; new N2, positive LN in two groups (LC+GC, or LC+EP, or GC+EP), regardless of number of involved lymph nodes; new N3, positive LN for all three groups (LC+GC+EP), regardless of number of involved lymph nodes. The new N classification was combined with the current parameter T, and a new TNM staging system was established.

### Statistical analysis

A 2-sided *P* value < 0.05 was considered to be statistically significant. The concordance between the two staging systems was computed by the inter-rater agreement analysis and non-weighted kappa value. The Kaplan-Meier method and long-rank test were used to compare survival curves. Univariate and multivariate analyses, computing *P* values and hazard ratios (HR) with 95% confidence interval (CI), were performed by using the Cox proportional hazards regression model, including in the multivariate analysis prognostic variables showing a P < 0.1 on univariate analysis. Multicollinearity among variables supposed to have high correlation was investigated with interaction analysis.

### Analysis of the prognostic performance of the two staging systems

We analyzed homogeneity (small differences in DFS time among patients classified into the same group by that system) by the likelihood ratio chi-square test, distinctiveness (patients classified into different groups have much greater differences in DFS time) and monotonicity (the mean DFS time in a more favorable stage is always longer than the DFS time in a poorer stage) by the linear trend chi-square test [26]. The multivariable logistic regression, within the best model defined by Cox’s analysis, was used to evaluate the predictive capability of the competing staging systems by computing the Bayesian information criterion (BIC) and the Harrell C-index [17,27]. The BIC is a criterion for model selection between two or more models; the model with the lowest BIC is preferred. The Harrell C-index measures the predictive accuracy of a model (a C-index of 1 indicates 100% predictive accuracy) and compares more models [18,28]. However, raw BIC values and Harrell C-index are unable to accurately quantify the improvement in risk prediction offered by a staging system and to assess its ability to discriminate patients who will develop the event of interest from patients who will not [5,29]. The most widely used method has been the analysis of the area-under-curve (AUC), calculated by the time-dependent receiver-operating-characteristic (ROC) curve for censored survival data. It estimates the probability that, at a certain time point, a recurrent patient is classified in a higher staging category than a patient who does not present with tumor relapse at that time [30,31]. Higher AUC values indicate better predictive ability. In addition, we applied, for all these analyses, the bootstrap-based technique that, by adding and removing patients from the original data for several times, and by computing the differences at each step, estimates the mean difference and its 95% CI. It allows to compare two different sets of data and, above all, to overcome limitations due to a small number of patients [32]. In brief, the distribution of the differences of the above mentioned indices for each staging system was gathered from 1000 bootstrap samples from the original data set, and mean values of the differences with their 95% CIs were computed. This method indicates a significantly different predictive ability of the two staging systems if the zero value is not included [17].

Statistical analysis was carried out using the SPSS 20.0 software (SPSS Inc., Chicago, IL, USA) and the statistical package R (version 3.2.5, R Foundation for statistical computing), integrated by the Medcalc^®^ software version 9.4.2.0 (Mariakerke, Belgium).

## Results

Table 1 summarizes patient characteristics. One hundred thirty-six patients (48%) had lymph node metastases, 57 of them in only one LN group (LC 29 patients, GC 28 patients); 36 patients had metastases in two LN groups (9 LC+GC, 15 LC+EP, and 12 GC+EP); 43 patients presented with LN metastases in all three groups; no patient was recorded with isolated positive nodes in the EP area.

**Table 1:** Characteristics and disease-free survival rate in 284 gastric cancer patient undergoing potentially curative surgery from 2000 to 2014 at Second University of Naples.

		Patients(%)	Recurrences(%)	5yrDFS	HR	95% CI *	*P* ^†^
Age (years) ^‡^	≤ 63	144(51)	50(35)	62.9	1	Referent	0.61
	> 63	140(49)	46(33)	62.0	1.10	0.60-1-35	
Gender	Male	170(60)	50(29)	67.9	1	Referent	0.07
	Female	114(40)	46(40)	54.0	1.43	0.96–2.14	
Serum CEA Levels (ng/mL)	≤ 3.5	228(80)	64(28)	69.8	1	Referent	< 0.001
	> 3.5	56(20	32(62)	33.7	2.55	1.67–3.90	
Performance Status	0	114(40)	36(32)	66.2	1	Referent	
	1	132(46)	48(36)	60.2	1.26	0.82–1.95	0.32
	2	38(14)	12(32)	55.8	1.58	0.82–3.05	
Tumor Site	Distal	112(39)	28(25)	72.1	1	Referent	
	Middle Third	104(37)	44(42)	53.0	2.04	1.27–3.27	0.013
	Upper Third	68(24)	24(35)	60.3	1.57	0.91–2.71	
Resection	Distal Gastrec.	78(27)	14(18)	78.7	1	Referent	0.001
	Total Gastrec.	206(73)	82(40)	56.2	2.56	1.45–4.52	
Macroscopic Type ^§^	Mass	48(17)	2(4)	95.2	1	Referent	
	Ulcerative	116(41)	32(28)	70.0	7.43	1.78–31.03	< 0.001
	Ulcer-Infiltrat.	92(32)	44(48)	47.6	12.84	3.11–53.00	
	Diff.-Infiltrat.	28(10)	18(64)	34.6	20.38	4.72–87.91	
Hystological Type ^║^	Differentiated	170(60)	48(28)	68.9	1	Referent	0.001
	Undifferent.	114(40)	48(42)	51.6	1.66	1.21–2.27	
Lauren's Classification	Intestinal	160(56)	44(27)	69.4	1	Referent	0.004
	Diffuse	124(44)	52(42)	53.0	1.81	1.21–2.71	
Tumor Depth (T)	1a	14(5)	0(0)	100.0			
	1b	32(11)	2(6)	92.9	1	Referent	
	2	58(20)	2(3)	96.2	0.50	09.71–3.59	
	3	56(21)	30(53)	42.5	12.08	2.88–50.59	< 0.001
	4a	112(39)	56(50)	43.5	10.85	2.64–44.50	
	4b	12(4)	6(50)	50.0	10.35	2.08–51.33	
Serosal Invasion	S-	160(56)	34(21)	76.6	1	Referent	< 0.001
	S+	124(44)	62(50)	43.9	2.89	1.90–4.40	
Node Stage (N)	0	148(52)	22(15)	83.2	1	Referent	
	1	52(18)	22(42)	55.8	3.14	1.74–5.68	
	2	48(17)	22(46)	43.4	3.56	1.97–6.44	< 0.001
	3a	22(8)	20(91)	9.1	10.86	5.86–20.13	
	3b	14(5)	10(71)	21.4	10.33	4,86–21.96	
New Node Stage (NewN)	0	148(52)	22(15)	83.2	1	Referent	
	1	57(20)	15(26)	73.3	1.79	0.93–3.45	< 0.001
	2	36(13)	20(55)	29.9	5.26	2.87–9.66	
	3	43(15)	39(91)	9.3	10.40	6.11–17.71	
Node Position ^¶^	None	148(52)	22(15)	83.2	1	Referent	
	LC	29(10)	7(24)	75.9	1.71	0.73–4.01	
	GC	28(8)	8(28)	70.6	1.86	0.83–4.20	
	EP	0(0)	0(0)	0	0.00	0.00–0.00	< 0.001
	LC+GC	9(4)	5(55)	44.4	5.73	2.17–15.16	
	LC+EP	15(6)	8(53)	25.4	4.44	1.97–9.98	
	GC+EP	12(5)	7(58)	22.9	6.23	2.65–14.63	
	LC+GC+EP	43(15)	39(91)	9.3	10.42	6.12–17.74	
TNM Stage	IA	40(14)	0(0)	100.0			
	IB	44(15)	2(4)	95.2	1	Referent	
	IIA	36(13)	6(17)	82.4	4.34	0.87–21.52	
	IIB	44(15)	14(32)	62.3	8.79	1.99–38.73	< 0.001
	IIIA	50(18)	26(52)	45.5	16.00	3.79–67.44	
	IIIB	50(18)	34(68)	27.9	24.89	5.97–103.78	
	IIIC	20(7)	14(70)	18.0	31.50	7.11–139.45	
New TNM Stage	IA	40(14)	0(0)	100.0			
	IB	45(16)	1(2)	97.7	1	Referent	
	IIA	35(12)	7(20)	78.8	10.71	1.31–87.11	
	IIB	48.17	12(25)	71.2	13.53	1.75–104.08	< 0.001
	IIIA	40(14)	20(50)	46.4	31.40	4.21–234.07	
	IIIB	55(19)	37(67)	25.7	55.05	7.54–401.73	
	IIIC	21(8)	19(90)	9.5	76.27	10.18–571.44	
No. of Resected Nodes ^‡^	≤ 27	150(53)	46(31)	66.3	1	Referent	0.31
	> 27	134(47)	50(37)	57.8	1.23	0.82–1.84	
PO Complications ^║^	No	222(78)	68(31)	66.8	1	Referent	< 0.001
	Yes	62(22)	28(45)	42.5	2.40	1.51–3.73	
PO Chemotherapy	No	102(36)	18(18)	79.8	1	Referent	0.001
	Yes	182(64)	78(43)	54.0	2.36	1.41–3.94	

CEA indicates carcinoembryonic antigen (normal value ≤ 3.5 ng/mL). Performance status according to the ECOG scale; postoperative complications were defined as grade II or higher of the Clavien-Dindo classification (ref. n° 33)

* 95% confidence interval

^†^ Kaplan-Meier method

^‡^ median value

^§^ according to Bormann's classification;

^║^ differentiated includes grading 1 and 2 tumors; undifferentiated includes grading 3 tumors and signet-ring cell cancers

^¶^ LC lesser curvature nodes (nodal groups 1, 3, and 5); GC greater curvature nodes (nodal groups 2, 4sa, 4sb, 4d, 6, and greater omentum); EP extra-perigastric nodes (nodal groups 7, 8a, 9, 10, 11p, 11d, and 12a)

The concordance among current and new lymph node categories, and among current and new TNM stages was good but not optimal, suggesting that the new staging system was different from the current classification (Tables 2 and 3). Indeed, more than one third of node positive patients (48 cases) were reclassified in different N and TNM stages. Specifically, 25 and 23 patients were moved into poorer categories and more favorable stages, respectively.

**Table 2:** 

Concordance between current N stage and new N stage						
	N0	N1	N2	N3a	N3b	Total(%)
new N0	148	0	0	0	0	148 (52.1)
new N1	0	41	16	0	0	57 (20.1)
new N2	0	11	18	4	3	36 (12.7)
new N3	0	0	14	18	11	43 (15.1)
Total (%)	148 (52.1)	52 (18.4)	48 (16.9)	22 (7.7)	14 (4.9)	284 (100)

At the end of each row is reported the total number (percentage) of patients belonging to that row. At the end of each column is reported the total number (percentage) of patients belonging to that column. Computed by inter-rater agreement analysis and k value (kappa value < 0.20 indicates poor agreement; kappa value > 0.80 indicates very good agreement): Concordance between current N stage and new N stage—k value = 0.685.

**Table 3:** 

Concordance between current TNM stage and new TNM stage								
	IA	IB	IIA	IIB	IIIA	IIIB	IIIC	Total (%)
new IA	40	0	0	0	0	0	0	40 (14.1)
new IB	0	43	2	0	0	0	0	45 (15.8)
new IIA	0	1	34	0	0	0	0	35 (12.3)
new IIB	0	0	0	42	6	0	0	48 (16.9)
new IIIA	0	0	0	2	32	6	0	40 (14.1)
new IIIB	0	0	0	0	12	34	9	55 (19.4)
new IIIC	0	0	0	0	0	10	11	21 (7.4)
Total (%)	40 (14.1)	44 (15.5)	36 (12.7)	44 (15.5)	50 (17.6)	50 (17.6)	20 (7.0)	284 (100)

At the end of each row is reported the total number (percentage) of patients belonging to that row. At the end of each column is reported the total number (percentage) of patients belonging to that column. Computed by inter-rater agreement analysis and k value (kappa value < 0.20 indicates poor agreement; kappa value > 0.80 indicates very good agreement): Concordance between current TNM stage and new TNM stage—k value = 0.801

### Analysis of tumor recurrence and comparison of prognostic performance

**Fig 1:**
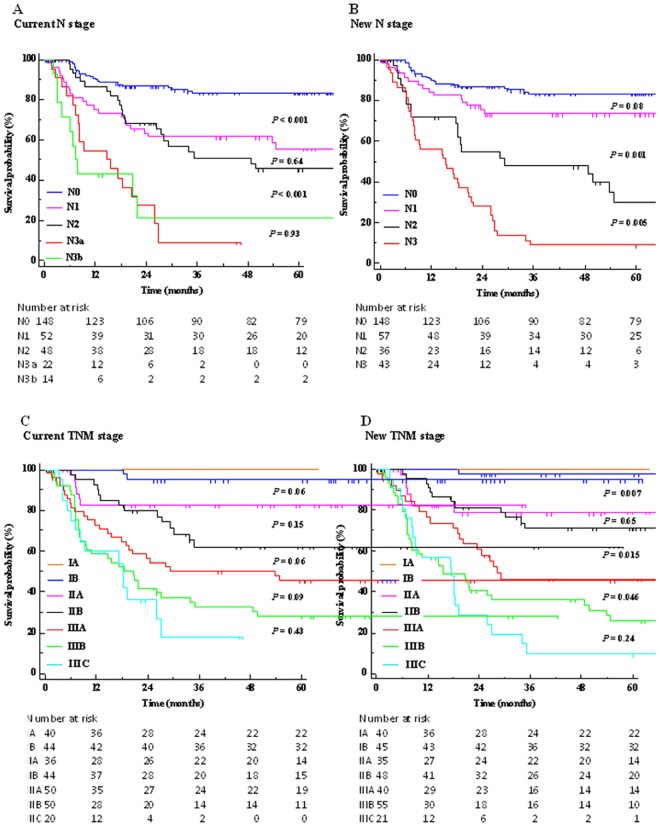
Disease-free survival rates for each staging system. (A) Current N stage. Shown are *P* values for pairwise comparisons of DFS curves in N0 *vs*. N1, N1 *vs*. N2, N2 *vs*. N3a, and N3a *vs*. N3b. (B) New N stage. Shown are *P* values for pairwise comparisons of DFS curves in new N0 *vs*. N1, N1 *vs*. N2, and N2 *vs*. N3. (C) Current TNM stage. Shown are *P* values for pairwise comparisons of DFS curves in different substages. (D) New TNM stage. Shown are *P* values for pairwise comparisons of DFS curves in different substages. **Note to figures 1C and 1D**: The iterations required to achieve significant DFS rates among different substages were eleven in the current TNM staging system (IA vs. IB, *P* = 0.207; IA vs. IIA, *P* = 0.007; IB vs. IIA, *P* = 0.060; IB vs. IIB, *P* < 0.001; IIA vs. IIB, *P* = 0.154; IIA vs. IIIA, *P* = 0.002; IIB vs. IIIA, *P* = 0.069; IIB vs. IIIB, *P* < 0.001; IIIA vs. IIIB, *P* = 0.096; IIIA vs. IIIC, *P* = 0.044; IIIB vs. IIIC, *P* = 0.433) and eight in the new TNM staging system (IA vs. IB, *P* = 0.388; IA vs. IIA, *P* = 0.003; IB vs. IIA, *P* = 0.006; IIA vs. IIB, *P* = 0.653; IIA vs. IIIA, *P* = 0.012; IIB vs. IIIA, *P* = 0.015; IIIA vs. IIIB, *P* = 0.046; IIIb vs. IIIC, *P* = 0.241).

**Fig 2:**
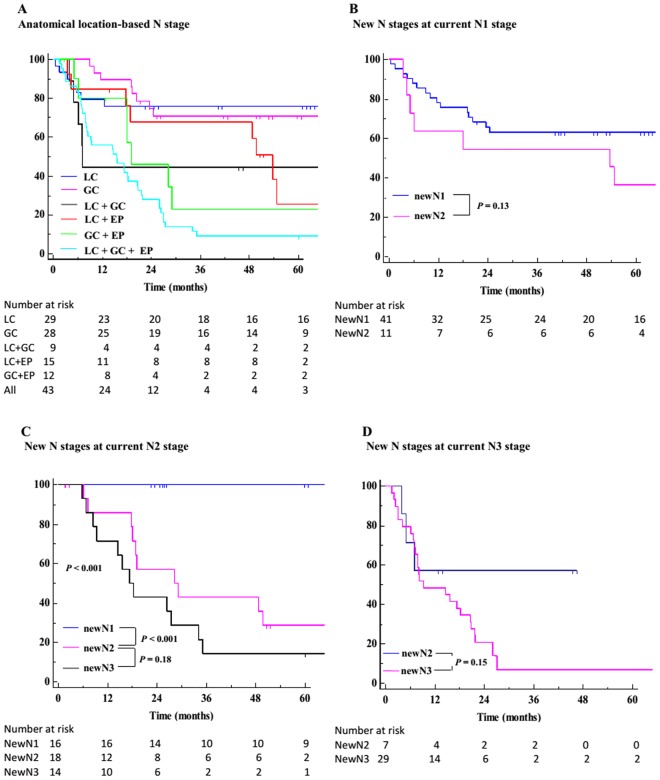
Disease-free survival rates for new N stages. (A) DFS rates among each combination of anatomical location-based lymph node groups [LC: lesser curvature; GC: greater curvature; EP: extraperigastric nodes]. (B) DFS rates for each new N stage at current N1 stage. Shown are *P* values for pairwise comparison of DFS curves in new N1 and N2. (C) DFS rates for each new N stage at current N2 stage. Shown are *P* values for pairwise comparisons of DFS curves in new N1 and N2, and N2 and N3. All curves *P* < 0.001. (D) DFS rates for each new N stage at current N3 stage. Shown are *P* values for pairwise comparison of DFS curves in new N2 and N3.

The 1- to 5-year overall survival rates were 88.5, 76.5, 70.2, 66.5, and 64.6%, respectively. At the end of the study, ninety-six patients (33.8%) presented tumor recurrence. The 1- to 5-year DFS rates were 81.5, 71.1, 64.5, 64.0, and 62.5%, respectively, and the time to recurrence ranged from 5 to 54 months (mean 15 ± 11, median 11 months). The 5-year overall survival rates according to the current and new TNM staging systems were 97.2 and 98.6% for stage I, 70.6 and 73.8% for stage II, and 32.6 and 27.8% for stage III, respectively. The new classification was demonstrated to have a significantly better homogeneity, discriminatory power, and monotonicity, and, overall, to display a better prognostic performance than the current staging system (Tables 4 and 5). On univariate analysis, the new staging model was demonstrated to be significantly related to cancer relapse (Table 1). For each substage, the new N and TNM classifications allowed a better patient stratification than the current staging system, with a reduced curve overlap during the whole follow-up time "Fig 1". In the current N stage, there was no significant difference between N1 and N2 patients; in addition, DFS rates were inverted and quite similar in N3a and N3b patients. On the contrary, the new N stage did not show divergences, and the recurrence rates were shown to significantly increase with worsening N categories. In addition, the new node classification was able to identify patients with different risks despite being currently grouped in the same node stage "Fig 2". The distribution of patients according to the current and new TNM staging systems is listed in Table 6. Interestingly, each new TNM stage (lower part of Table 6) should have included patients with different current stages but without significant differences in 5-years DFS rates; on the contrary, the current TNM stages (upper part of the Table 6) included patients with different new stages and with different DFS rates (significant differences in all stages, with the exception of stage IIA). In addition, as listed in the note to "Fig 1C and 1D", to achieve significant DFS rates among different substages, the current TNM system required eleven iterations; on the contrary, only eight comparisons were necessary within the new TNM model, suggesting a better stratification.

**Table 4:** Prognostic performance between the current and new nodal and TNM stages.

	pN Stage	New N Stage	TNM Stage	NewTNM Stage
Likelihood Ratio *	71.71	97.15	103.25	126.55
Linear Trend ^†^	60.76	90.53	84.36	101.91

* chi-square test; higher values show better homogeneity (small difference in DFS time among patients classified into the same group by this system)

^†^ chi-square test; higher values mean better discriminatory power (patients classified into different groups have much greater differences in DFS time) and monotonicity (the mean DFS time in a more favorable stage is always longer than the DFS time in a poor stage)

**Table 5:** Prognostic performance between the current and new nodal and TNM stages. Bootstrap analysis.

Bootstrap analysis for N parameter *					
	mean ± SD		Difference		
	pN Stage	new N Stage	mean ± SD	95% CI ^†^	*P* value ^‡^
Likelihood Ratio ^§^	67.60 ± 13.13	91.83 ± 19.19	24.22 ± 7.80	18.50–29.94	< 0.001
Linear Trend ^║^	57.97 ± 8.55	86.39 ± 13.60	28.42 ± 6.89	23.49–33.35	< 0.001
Bootstrap analysis for TNM stage ^‡^					
	mean ± SD		Difference		
	TNM Stage	newTNM Stage	mean ± SD	95% CI ^†^	*P* value ^‡^
Likelihood Ratio ^§^	101.80 ± 4.30	122.74 ± 8.54	20.94 ± 6.70	16.13–25.74	< 0.001
Linear Trend ^║^	82.63 ± 3.28	98.09 ± 6.75	15.46 ± 6.05	11.13–19.79	< 0.001

* 1000 samples

^†^ 95% confidence interval

^‡^ independent samples t-test

^§^ chi-square test; higher values show better homogeneity (small difference in DFS time among patients classified into the same group by this system)

^║^ chi-square test; higher values mean better discriminatory power (patients classified into different groups have much greater differences in DFS time) and monotonicity (the mean DFS time in a more favorable stage is always longer than the DFS time in a poor stage)

**Table 6:** Patient distribution according to the TNM 7th edition and the new staging system.

Stage	New Stage	Patients n.	Recurrences n.	5yr-DFS	*P* value
IA	IA	40	0	100.0	—
	Global	40	0	100.0	
IB	IB	43	1	97.6	
	IIA	1	1	0.0	< 0.001
	Global	44	2	95.2	
IIA	IB	2	0	100.0	
	IIA	34	6	81.3	0.52
	Global	36	6	82.4	
IIB	IIB	42	12	66.2	
	IIIA	2	2	0.0	0.045
	Global	44	14	62.3	
IIIA	IIB	6	0	100.0	
	IIIA	32	18	40.2	0.054
	IIIB	12	8	33.3	
	Global	50	26	45.5	
IIIB	IIIA	6	0	100.0	
	IIIB	34	26	18.8	0.013
	IIIC	10	8	20.0	
	Global	50	34	27.9	
IIIC	IIIB	9	3	66.7	
	IIIC	11	11	0.0	0.045
	Global	20	14	18.0	
New Stage	Stage	Patients n.	Recurrences n.	5yr-DFS	*P* value
IA	IA	40	0	100.0	—
	Global	40	0	100.0	
IB	IB	43	1	97.6	
	IIA	2	0	0.0	0.82
	Global	45	1	97.7	
IIA	IB	1	1	100.0	
	IIA	34	6	81.3	0.10
	Global	35	7	78.8	
IIB	IIB	42	12	66.2	
	IIIA	6	0	100.0	0.11
	Global	48	12	71.2	
IIIA	IIB	2	2	0.0	
	IIIA	32	18	40.2	0.69
	IIIB	6	0	100.0	
	Global	40	20	46.4	
IIIB	IIIA	12	8	33.3	
	IIIB	34	26	18.8	0.07
	IIIC	9	3	66.7	
	Global	55	37	25.7	
IIIC	IIIB	10	8	20.0	
	IIIC	11	11	0.0	0.09
	Global	21	19	9.5	

### Comparison of the predictive capability of the staging systems

**Fig 3:**
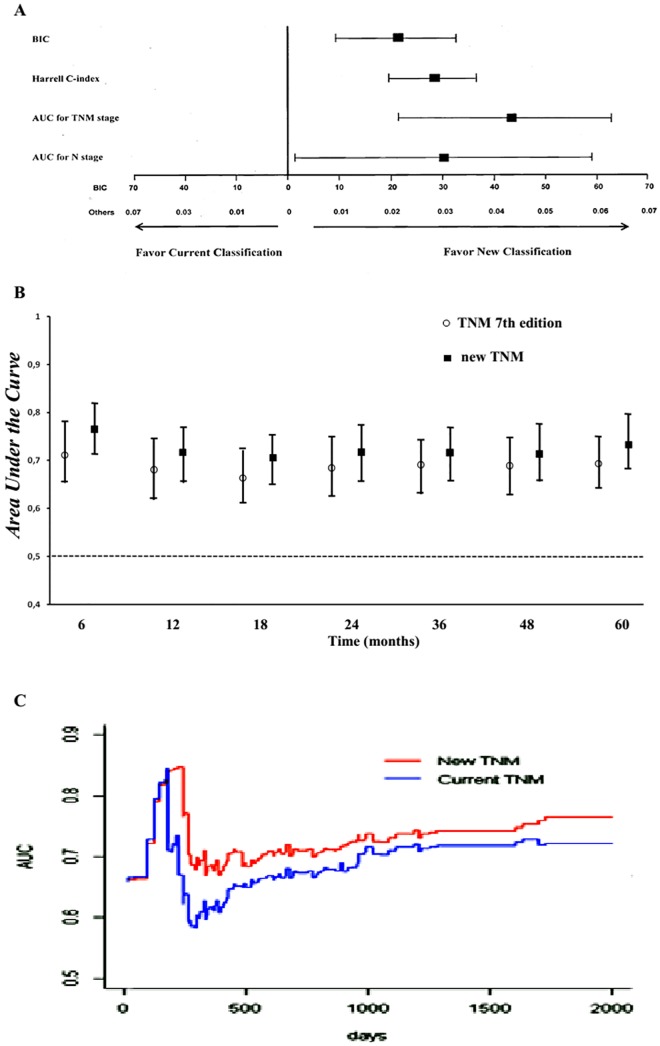
Accuracy of the competing staging systems to predict disease-free survival rate. (A) Results from bootstrap analysis (1,000 samples). The mean differences in BIC and Harrell C-index with 95% confidence intervals were based on multivariable logistic regression including variables grouped in the best model selected by Cox's analysis. The mean differences in the Area Under the Curve (AUC) for TNM stage and AUC for N stage with 95% confidence intervals were calculated by using the time-dependent receiver-operating-characteristic (ROC) analysis for censored survival data. By these procedures, 95% CIs were computed for differences in the four indices indicating significantly different predictive ability of two staging systems if the zero value was not included. (B) Areas under the ROC curves computed by the time-dependent analysis for censored survival data based on different staging systems according to TNM 7th edition and the new TNM model. Mean AUC values and their 95% CIs were calculated at several time points of follow-up and for each competing system. Error bars represent 95% bootstrap confidence intervals. (C) Analysis of the predictive accuracy of the two competing staging systems through the 5th year of follow-up, computed by the time-dependent ROC analysis for censored survival data.

The best model fitting tumor recurrence included, as independent prognostic variables, elevated preoperative serum CEA levels, total gastrectomy, serosal invasion, lymph node metastases, occurrence of postoperative complications [33], and both TNM staging systems, with the new model showing an almost doubled HR compared with the current TNM stage (Table 7). In addition, the interaction analysis excluded any correlation. In this model, the bootstrap-based BIC mean values were 274 (95% CI 263–285, range 251–298) and 253 (95% CI 248–258, range 242–265) for current and new TNM staging systems, respectively. The difference was 21 and 95% CI difference was 14–28 (*P* = 0.001 "Fig 3A"). The new TNM staging system showed a higher Harrell C-index (mean 0.91, 95% CI 0.91–0.92, range 0.87–0.90) than the current TNM model (mean 0.88, 95% CI 0.88–0.89, range 0.87–0.90). The difference was 0.02, 95% CI difference was 0.02–0.03, *P* < 0.001 "Fig 3A".

**Table 7:** Multivariate analysis related to disease-free survival in 284 gastric cancer patients undergoing potentially curative surgery. Cox's proportional-hazards model.

	Coefficient	Standard Error	Hazard Ratio	95% CI * Hazard Ratio	*P* value
Sex (female)	0.370	0.231	1.44	0.91–2.27	0.110
Serum CEA level (>3.5ng/mL)	0.704	0.239	2.02	1.26–3.23	**0.003**
Tumor Site (Up/Mid Third)	0.009	0.288	1.01	0.57–1.77	0.974
Type of Resection (Total)	0.888	0.366	2.43	1.18–4.97	**0.015**
Macroscopic Type (Infiltrative)	0.407	0.236	1.50	0.94–2.38	0.085
Histological Type (Undifferent.)	0.520	0.379	1.68	0.80–3.53	0.170
Lauren (Diffuse)	0.643	0.397	1.90	0.87–4.13	0.105
Tumor Depth (> 2)	0.531	0.244	1.70	1.05–2.74	**0.030**
Node Stage (N > 0)	1.617	0.278	5.04	2.92–8.68	**0.001**
Current TNM Stage (> IIB)	1.217	0.399	3.37	1.54–7.38	**0.002**
New TNM Stage (>IIB)	1.833	0.409	6.25	2.80–13.92	**0.001**
PO Complications (Yes)	1.200	0.270	3.32	1.95–5.63	**0.001**
PO Chemotherapy (Yes)	0.059	0.315	1.06	0.57–1.96	0.852

* 95% confidence interval

Statistical assessment of the predictive performance of the two staging systems, when computing the AUC values with survival ROC analysis, demonstrated a quite substantial efficacy of both models (bootstrap-based mean AUC values between 0.667 and 0.768). However, it revealed a constant superiority of the new classification system over time "Fig 3B". The global 5-year AUC values were 0.696 ± 0.02 (95% CI 0.681–0.710, range 0.667–0.722) and 0.738 ± 0.02 (95% CI 0.722–0.755, range 0.697–0.768) for current and new TNM staging system, respectively. The mean difference was 0.042 ± 0.001 and 95% CI difference was 0.022–0.063 (*P* < 0.001), thus showing that the new TNM staging model had a significantly better prognostic accuracy than the current TNM classification "Fig 3A". In addition, AUC values were 0.519 ± 0.02 (95% CI 0.498–0.539, range 0.487–0.571) and 0.549 ± 0.03 (95% CI 0.524–0.573, range 0.498–0.588), for current and new lymph node staging system, respectively. The mean difference was 0.030 ± 0.014 [95% CI difference was 0.001–0.059 (*P* = 0.043)], thus demonstrating that the new model performed better than the old model "Fig 3A". Comparison of time-dependent ROC curves for both models is depicted in "Fig 3C". The greatest difference between the two curves was evident between the sixth and fourteenth month after surgery, which was the period with the highest risk of cancer recurrence; at this time, the new TNM staging system was shown to possess a significantly better accuracy than the old model. After that time frame, the curves remained separate, with continuous increments in prognostic performance.

## Discussion

This study shows that the anatomical location-based classification of involved lymph nodes was significantly associated with prognosis and long-term outcome in resected gastric cancers. The predictive capability and prognostic accuracy of the new staging system were better than those shown by the current system. It would suggest that the anatomical extent of lymph node metastasis should be considered for adequate gastric cancer patient classification. These results fully address a recent Eastern experience promoting the implementation of this new staging system [18].

The first editions of AJCC TNM cancer staging manual adopted an anatomical-based system for N category [8]. With time, this classification seemed to be not so accurate and somewhat complex; therefore, from the 5th edition on, the nodal stage was completely transformed into a numeric-based system [9]. However, the latest edition highlighted the need for a further change since the correct definition of the parameter N was still far from a definitive standardization [8,10]. Several reports, mostly from Asia, have investigated the prognostic performance of the different AJCC editions, with discordant results [26,34–39]. A Western experience is lacking. To date, only three studies have investigated this issue. Warneke et al [7] concluded that the 7th edition did not improve the predictive ability of the previous one; they proposed a revised model, which, however, failed to obtain external validation [5]. Another European study was in favor of the superiority of the latest edition, although a statistical comparison between the recent staging systems was not carried out, a common pitfall in many other studies [40]. Alternative tools present critical issues as well; for example, the cut-off point of lymph node ratio has not yet been validated [5,41,42]. Thus, the correct classification of node involvement in gastric cancer remains controversial. In addition, current models based on numeric N staging are devoid of information regarding both the anatomical extent of the disease and lymphadenectomy and the involvement of proximal or distant nodes.

Recently, Choi et al [18] proposed a new lymph node involvement classification based on anatomical site rather than on number of metastatic nodes. This new system proved to be simple, intuitive, easy to apply, and well-performing. It was proposed as a reliable alternative to the current TNM staging system, upon Western world validation. To this end, we have reclassified 284 non-metastatic gastric cancers undergoing potentially curative surgery and evaluated risk prediction with the two competing staging models by means of rigorous statistical analyses [29–31]. The new categories differed from those calculated with the current system, and more than one third of node positive patients were reclassified in different stages. Interestingly, no patient had isolated extraperigastric positive nodes (skip metastasis). Although this may suggest a sampling error, it should be considered that a very low incidence rate of skip metastasis has nonetheless been reported worldwide [43]. The new categories were associated with recurrence and DFS rates. Overall, a greater prognostic performance than that of the actual staging system was recorded. Interestingly, the patients currently grouped as N1 (1–2 metastatic nodes) and N2 (3–6 positive LNs) displayed no significant differences in DFS rates. It would suggest that, in the 7th edition, the division into two categories of the N1 stage of the 6th edition has not translated into improved outcome, a critical factor already noticed by others in larger series [5]. In addition, N3b patients did better than N3a patients and no significant differences were observed between the two substages, an intriguing but seldom reported issue due to frequent unification of the two categories [36]. On the contrary, the new N stage demonstrated homogeneity and monotonicity, with statistical differences among all categories [44]. Impressively, the most intriguing finding was the observation of a worsening prognosis with more advanced new N stages, despite the same current N stage. It would indicate that the anatomical location-based classification of lymph node metastasis is an important factor for gastric cancer prognosis. Besides, the number of iterations required to achieve significance among all substages was lower with the new TNM model.

Our study was supported by a robust and reliable statistical analysis. In this regard, the current N and TNM classifications were shown to possess a significantly worse prognostic accuracy than the new model, thus suggesting a need for revision. Of note, the new TNM system seemed to be particularly effective in the early phase of follow-up, when the greatest number of tumor recurrences occur [17,26]. Additional advantages of the new model are represented by a better surgical accuracy during lymphadenectomy, since extraperigastric LN dissection must be performed to stage the tumor; a simpler pathologic examination, since three LN groups are required for evaluation; no increase in the number of substages, which could lead to higher complexity [7]. Our study has some limitations. First, the sample size was too small to draw definitive conclusions, although it should be remembered that other studies analyzed quite similar number of patients too [34,35,44]. However, our results were backed by the bootstrap method. This procedure provides a way of quantifying the uncertainties in the inferences that can be drawn from a sample of data, and *de facto* overcomes limitations due to small numbers of patients [17,32]. Second, this was a retrospective study and, as such, had a significant risk of inherent confounding bias [45]. In addition, we were aware of the difficulties in reclassifying patients in case different nodal groups had not been precisely identified by pathologists. However, all patients were enrolled with stringent eligibility criteria and no patient was lost at follow-up. The reclassification of our patients was very easy and quick because, as stated elsewhere [6], our policy has always been to carefully isolate each nodal station to be sent separately to pathological examination, in order to meticulously document positive and negative nodes for each individual nodal station [15,46].

## Conclusion

Our results seem to validate the new staging system proposed by Choi et al [18] based on the anatomical location of metastatic lymph nodes in radically resected gastric cancer patients. In an era in which multimodal therapy and patient-tailored therapy are crucial, optimal prognostic accuracy is mandatory to define the best treatment. These results could aim at influencing a future revision of the AJCC edition [47,48]. To the best of our knowledge, our study is the first from the Western world to compare nodal stage based on anatomical location of metastatic nodes rather than on the number of positive nodes. Further studies involving a greater number of cases may be necessary to confirm the definitive applicability of this staging system.
